# Quantifying person-level brain network functioning to facilitate clinical translation

**DOI:** 10.1038/tp.2017.204

**Published:** 2017-10-17

**Authors:** T M Ball, A N Goldstein-Piekarski, J M Gatt, L M Williams

**Affiliations:** 1 Department of Psychiatry and Behavioral Sciences, Stanford University School of Medicine, Stanford, CA, USA; 2 Neuroscience Research Australia, Sydney, NSW, Australia; 3 School of Psychology, University of New South Wales, Kensington, NSW, Australia

## Abstract

Although advances in neuroimaging have yielded insights into the intrinsic organization of human brain networks and their relevance to psychiatric and neurological disorders, there has been no translation of these insights into clinical practice. One necessary step toward clinical translation is identifying a summary metric of network function that is reproducible, reliable, and has known normative data, analogous to normed neuropsychological tests. Our aim was therefore to establish the proof of principle for such a metric, focusing on the default mode network (DMN). We compared three candidate summary metrics: global clustering coefficient, characteristic path length, and average connectivity. Across three samples totaling 322 healthy, mostly Caucasian adults, average connectivity performed best, with good internal consistency (Cronbach’s *α*=0.69–0.70) and adequate eight-week test–retest reliability (intra-class coefficient=0.62 in a subsample *N*=65). We therefore present normative data for average connectivity of the DMN and its sub-networks. These proof of principle results are an important first step for the translation of neuroimaging to clinical practice. Ultimately, a normed summary metric will allow a single patient’s DMN function to be quantified and interpreted relative to normative peers.

## Introduction

Intrinsic brain networks have been extensively examined in groundbreaking basic neuroscience research,^1, 2, 3^ and have shown relevance to psychiatric disorders in case-control studies.^4, 5^ However, there has been no translation of these insights into clinical practice.^6^ One major barrier to clinical translation is that we do not yet have a way to quantify and interpret a single patient’s network function.^7, 8^ Our aim was therefore to establish the proof of principle for a reliable summary metric that can quantify the intrinsic network function of a single patient and allow it to be interpreted relative to healthy normative peers.

Non-invasive human neuroimaging such as functional magnetic resonance imaging (fMRI) allows intrinsic brain networks to be examined using functional connectivity (that is, by identifying brain regions with temporally correlated activity). Currently, a major analytic approach to functional connectivity is to examine pairwise functional connectivity between a seed region of interest and the rest of the brain.^3^ However, this process is not standardized, requiring each researcher to select and define the seed region and set statistical and spatial thresholds for significant connectivity. Each of these steps tends to differ from researcher to researcher and even study to study, making it difficult to compare across studies to understand normal or abnormal network function. This variability also makes false-positive results more likely^9^ and can result in over-fitting due to the high dimensionality of neuroimaging data.^10^

A related issue with this analytic approach is that it is easy to interpret group differences in a specific pair of regions as representing group differences in the entire network. In other words, when functional connectivity between one pair of regions differs between cases and controls, a common inference is that this is characteristic of an entire network’s dysfunction.^11, 12, 13^ However, it may also be that the observed group difference applies only to the specific pair of regions and not to the network per se. In order to differentiate these two interpretations and to reduce false-positive findings, a standard metric that summarizes connectivity within a whole network is needed.

Our aim was therefore to identify a standardized summary metric for functional connectivity in a specific network. In order to facilitate clinical translation, we focused on aggregating across all pairwise connectivity within a network. We recognize that independent components analysis (ICA) is a valid exploratory statistical technique that has some benefits over the pairwise approach.^14, 15, 16^ However, because ICA involves re-defining the networks of interest based on each specific subject or group of subjects, it is not suited to our goal of a straightforward metric that can be readily translated into clinical practice.^17^

The optimal summary metric must satisfy several criteria.^7^ First, it must define a network in a standardized manner across subjects (not newly defining each time as in ICA) and systematically include all connections within a network (not only connections to a single seed region). Second, it must reliably measure network connectivity within a single imaging session (that is, have adequate internal consistency) and across sessions within the same person (that is, have adequate test–retest reliability). In other words, the metric should be robust to subtle variations in mood and fatigue over time (although it may also be of separate interest to identify features of networks that do vary with these state differences, that is not the purpose here). Third, key properties of the metric, such as its mean and standard deviation, should replicate across samples. Fourth, in order for a summary metric to meaningfully communicate information about a specific individual it must have known normative data. Finally, the metric must balance the competing ideals of capturing the complexity of brain networks on one hand, and ease of use and interpretation on the other.

**Figure 1:**
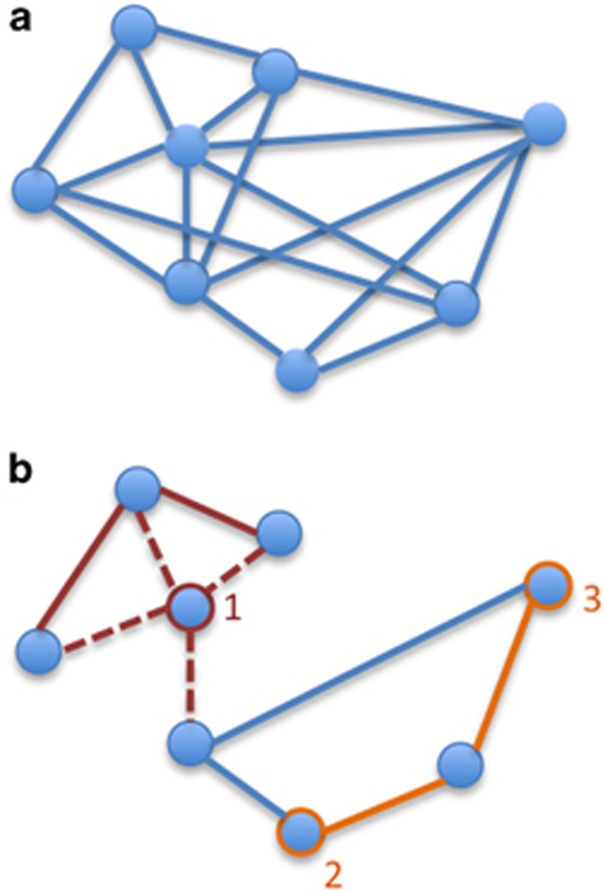
Illustration of candidate summary metrics. (**a**) Average connectivity is the average magnitude of all pairwise functional connectivity values (note that a subset of connections is shown in the figure for clarity). (**b**) Topological properties are derived from a representation of the network with a proportion of the weakest connections set to zero (represented by lines present in **a** and absent in **b**). The clustering coefficient of node 1 is the weighted proportion of connections among that node’s neighbors. Neighbors of node 1 are illustrated in the figure by red dashed lines, connections among those neighbors by red solid lines. The global clustering coefficient is the aggregate of the clustering coefficient for all nodes in the network and indicates the network’s short-range efficiency. The path length between nodes 2 and 3 is the weighted shortest distance between those nodes, illustrated in the figure by orange solid lines. The characteristic path length is the average shortest distance across all pairs of nodes in the network and indicates the network’s long-range efficiency.

In order to identify the metric that best balances these competing ideals, we compared the complex topological properties of global clustering coefficient and characteristic path length^18, 19, 20^ to a simple average of functional connectivity strength (Figure 1). The global clustering coefficient captures the short-range efficiency of a network by measuring the probability that two regions with high functional connectivity to a third region also have high functional connectivity to each other. The characteristic path length captures the long-range efficiency of a network by measuring the shortest number of connections between two regions, on average across the network. Average functional connectivity captures overall strength of connectivity within a network, providing a parsimonious, easily interpretable and easy to use summary metric.

We focus here on comparing these metrics within the default mode network (DMN), which has key nodes in the posterior cingulate cortex, medial prefrontal cortex and angular gyrus that are coherent at rest and deactivated in concert during tasks.^2, 21, 22, 23^ To define the DMN we used a previously validated and openly available cortical parcellation scheme that was developed based on consistent patterns of resting functional connectivity.^21^ We focused on the DMN because it is one of the most well established intrinsic networks, and one with arguably the clearest relevance for a broad range of psychiatric and neurological conditions.^4, 24^ The DMN has been implicated in self-reflective and interoceptive functions, and seminal case-control studies show that healthy adults have characteristically different DMN connectivity than those with psychiatric conditions.^4, 25, 26^ However, variability in these findings reflects the typically small sample size as well as the methodological and conceptual issues discussed above, highlighting the need for a summary metric that can characterize individual patients (not just groups of patients).

We evaluated three candidate summary metrics for the DMN: global clustering coefficient, characteristic path length and average connectivity. For each metric, we examined the internal consistency (Cronbach’s *α*, with>0.7 considered adequate^27^) and test–retest reliability (intra-class coefficient, with >0.6 considered moderate and >0.8 considered substantial^28^). We also examined the reproducibility of the mean values, standard deviations, and internal consistency across three samples (test–retest reliability could only be computed in Sample 3): Samples 1 and 2 consist of 255 healthy Caucasian adult twins with fMRI data (65% monozygotic, 33% dizygotic and 2% unknown zygosity), divided such that each twin pair was split across the two samples,^29^ and Sample 3 consists of 67 healthy adults that were recruited as controls for an antidepressant medication trial.^30^ Table 1 describes the demographic characteristics of each sample. We also provide normative data from the full group of 322 healthy adults and illustrate the potential for clinical use of such norms with a case example.

We first compared the two topological properties against average connectivity strength. We then examined how the best metric performed in the whole DMN relative to two theoretically important sub-networks:^1, 31^ (i) an anterior sub-network comprised of connections within medial prefrontal cortex (PFC) regions and (ii) a posterior-to-anterior sub-network comprised of connections between posterior (that is, posterior cingulate cortex; PCC) and anterior (that is, medial PFC) midline cortical regions.

## Materials and methods

### Study design

This study aimed to identify a standardized summary metric for functional connectivity in the DMN. The design was cross-sectional and observational, and involved three samples of healthy adult volunteers. Sample sizes and inclusion/exclusion criteria were determined a priori and publically specified.^29, 30^ The first two samples comprised 270 healthy Caucasian adult twins,^29^ divided such that each twin pair was split across the two samples. After exclusions for incomplete fMRI data (*n*=7), fMRI data acquisition errors (*n*=2), poor fMRI data quality (*n*=4), or participants withdrawn from the study (*n*=2), Sample 1 consisted of 126 healthy adults and Sample 2 consisted of 129 healthy adults. Sample 3 comprised 69 healthy adults that were recruited as controls for an anti-depression medication trial (^30^: clinicaltrials.gov identifier NCT00693849), with one participant excluded for incomplete fMRI data and another for poor fMRI data quality, leaving 67 in the analysis (*n*=65 for test–retest analysis due to *n*=2 missing data at eight-week follow-up). Table 1 shows the demographic characteristics of these samples. Note that sample sizes were reduced for the internal consistency analysis due to requirements for amount of useable fMRI data in each quintile of the time series (Sample 1 *n*=87 with sufficient data in all quintiles, Sample 2 *n*=98, Sample 3 *n*=38). All participants provided written informed consent in accordance with approval from the Human Research Ethics Committee of the University of Sydney.

### Image acquisition

The same imaging acquisition parameters were used for all samples. MRI images were acquired with a 3.0-T GE Signa scanner and an eight-channel head coil in Sydney, Australia. The scan consisted of five tasks and a 3D T1-weighted structural MRI scan. MR images for each task were acquired using echo planar imaging (TR=2500 ms, TE=27.5 ms, matrix=64 × 64, FOV=24 cm, flip angle=90 degrees). Forty slices, each 3.5 mm thick, covered the whole brain in each volume. For each task, 120 volumes were collected with a total scan time of 5 min and 8 s. The details of the five tasks have been previously described.^32^ Briefly, tasks assessed (i) selective attention using an auditory oddball task, (ii) working memory using a continuous performance task, (iii) inhibition processes using a Go-NoGo task and (iv) conscious and (v) non-conscious processing of emotional faces. Functional connectivity was derived from the residual time series when all five tasks were concatenated, following the removal of task and covariate effects (more details below). This procedure results in patterns of functional connectivity that closely mimic those found in resting state scans,^33^ and can also be considered to assess the “task negative” nature of the DMN.^2^

Structural MRI 3D T1-weighted images were acquired in the sagittal plane using a 3D spoiled gradient echo (SPGR) sequence (TR=8.3 ms; TE=3.2 ms; flip angle=11 degrees, TI=500 ms, NEX=1, ASSSET=1.5, matrix=256 × 256). A total of 180 contiguous slices, each 1 mm thick, covered the whole brain with an in-plane resolution of 1 mm x 1 mm. All participants were instructed to refrain from caffeine and tobacco use prior to the scan.

### Image preprocessing

The same preprocessing and data analysis steps were conducted on all samples. Preprocessing and data analysis were performed using Statistical Parametric Mapping software implemented in MATLAB (SPM8; Wellcome Department of Cognitive Neurology, London, UK). First, images were motion corrected and un-warped using default parameters in SPM8. Next, time points with large head movements or extreme changes in blood-oxygenation level dependent (BOLD) signal intensity were identified and censored (that is, temporally masked) from the analysis using the time series difference analysis toolbox (http://www.fil.ion.ucl.ac.uk/spm/ext/#TSDiffAna). Large head movement was defined as frame-wise displacement from one time point to the next of >0.3mm, calculated as the sum of the absolute values of the differentiated realignment estimates.^34^ Extreme changes in signal intensity were calculated as the mean squared difference in signal intensity from one time point to the next divided by the mean signal across the volume; scaled signal intensity differences of greater than 10 were censored. A temporal mask was then created for each censored time point plus the subsequent time point and used as regressors of no interest in the first-level statistical models described below.^34, 35^ Since movement related artifacts have been shown to impact data acquired before and several seconds the movement,^34^ a total of four temporal masks were created for each movement spike (an additional volume before and two volumes after the movement spike). Images were then slice time corrected, spatially normalized to Montreal Neurological Institute (MNI) space^36^ and smoothed using an 8 mm full-width-at-half-maximum Gaussian kernel in SPM8.

### Computation of functional connectivity

For each fMRI task, the BOLD responses for each experimental condition were modeled in the general linear model framework for a first-level (that is, single subject) statistical model (see Korgaonkor *et al.*^32^ for details). Motion effects were also modeled using the Volterra expansion of the realignment parameters, yielding 24 regressors. Additional covariates of non-interest for each task included the mean signal time course extracted from ventricle and white matter masks as well as the temporal masks derived from the volume censoring described above. The resting state signal was then extracted based on the concatenated time series across the entire scan session, using the residual after removing the above task and covariate effects. Finally, a band-pass filter (0.009 Hz<*f*<0.08 Hz) was applied.

In order to ensure sufficient data for a reliable functional connectivity estimate, any subject with fewer than 300 un-censored time points was excluded from the analysis (*n*=2 in Sample 1, *n*=2 in Sample 2, *n*=1 in Sample 3). For the internal consistency reliability analysis in which the time series was divided into quintiles, a minimum of 100 un-censored time points was required in each quintile. This relatively liberal threshold was selected to enhance the generalizability of results to settings or populations with only moderate data quality.

Finally, resting functional connectivity between nodes of the DMN was computed using the Data Processing & Analysis of Brain Imaging toolbox.^37^ First, each subject’s cortex was parcellated into 333 regions, 41 of which comprise the DMN.^21^ Second, Pearson correlations were computed for the average time courses of all pairwise regions within the DMN, yielding a 41 by 41 matrix of functional connectivity. The magnitude (that is, absolute value) of the Fisher*Z* transformed correlation was used for all subsequent analyses.

### Computation of summary metrics

We compared the complex topological properties global clustering coefficient and characteristic path length^18, 19, 20^ to a simple average of functional connectivity strength. Topological properties (weighted global clustering and weighted characteristic path length) were computed using the *Tnet* package in R Statistics.^38^ The 41 by 41 functional connectivity matrix described above was converted to a non-directed weighted graph with edge weights defined as the functional connectivity between two nodes. Three graphs were created for each subject, each using a different threshold below which edge weights were set to zero. Thresholds were selected so that 70, 75 or 80% of all edges were retained, because 70% was the minimum threshold such that all nodes had at least one edge for all participants in Sample 1. Because the three thresholds performed similarly, results from the less stringent thresholds (75 and 80% edges retained) are reported in the Supplementary Materials.

Average DMN connectivity was defined as the average magnitude (that is, absolute value) of Fisher Z transformed functional connectivity across the 41 by 41 matrix of DMN regions. Average connectivity in the anterior sub-network was defined as the average magnitude (that is, absolute value) of Fisher Z transformed functional connectivity across 14 DMN regions whose centroid coordinate is within 15 mm of the midline in the frontal lobe. Average connectivity in the posterior-to-anterior sub-network was defined as the average magnitude (that is, absolute value) of Fisher Z transformed functional connectivity between the posterior DMN (two DMN regions whose centroid coordinate is within the PCC) and the anterior DMN (14 DMN regions whose centroid coordinate is within 15 mm of the midline in the frontal lobe). Networks and sub-networks were visualized using BrainNet Viewer (http://www.nitrc.org/projects/bnv,^39^).

### Evaluation of potential summary metrics

In each sample, distributional features (that is, mean, standard deviation) and internal consistency reliability were computed for all potential summary metrics. Internal consistency reliability was computed by estimating resting state connectivity based on quintiles of the full time series, corresponding to a separate estimate for each task period, and computing Cronbach’s *α* based on the quintiles. Potential summary metrics were also evaluated based on replication, defined as a non-significant difference of means across the three samples, and a Cronbach’s *α*value in Sample 2 or Sample 3 falling within the 95% confidence interval of the value from Sample 1. Finally, Sample 3 also allowed for an evaluation of test–retest reliability (eight weeks between identical scans) using agreement intra-class coefficient (ICC).

## Results

### Comparison between three candidate metrics

We first examined the internal consistency, test–retest reliability, and reproducibility across samples of the global clustering coefficient, characteristic path length, and average connectivity. The results are summarized in Table 2.

Internal consistency was adequate for average connectivity but low for global clustering and characteristic path length. Test–retest reliability over eight weeks (available only in Sample 3) was moderate for average connectivity but low for global clustering and characteristic path length. Means, standard deviations, and internal consistency reliability in Samples 2 and 3 reproduced the values found in Sample 1 for global clustering and average connectivity. Internal consistency for characteristic path length in Sample 3 was outside the Sample 1 95% confidence interval and thus considered a lack of replication for this metric. Overall, average connectivity was more reliable than global clustering or characteristic path length, and was used for the subsequent analyses. Notably, test–retest reliability values for average connectivity were greater than those based on voxel-wise connectivity analyses with shorter test–retest intervals,^40^ and comparable to those based on ICA.^16^

### Sub-network averages

**Figure 2:**
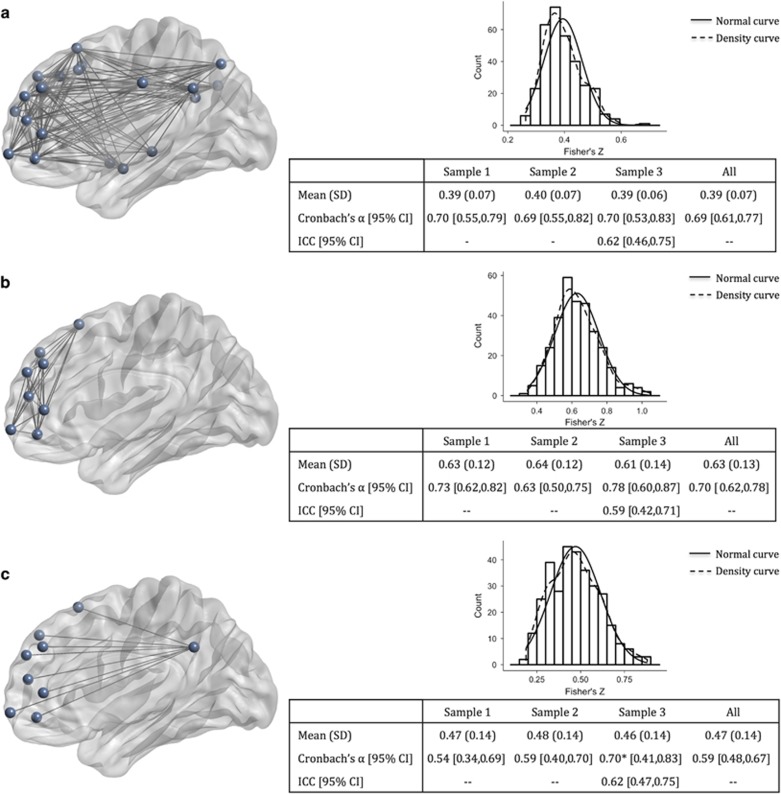
Replicability, internal consistency, and test–retest reliability of average functional connectivity. Means and standard deviations are in units of Fisher’s *Z*. (**a**) Whole network: average of all pairwise connections between all DMN regions. (**b**) Anterior sub-network: average of all pairwise connections between medial PFC regions within the DMN. (**c**) Posterior-to-anterior sub-network: average of all connections between PCC regions and medial PFC regions within the DMN. CI, confidence interval; DMN, default mode network; ICC, intra-class correlation coefficient; PCC, posterior cingulate cortex; PFC, prefrontal cortex; SD, standard deviation. *indicates value outside 95% CI of Sample 1.

We next examined average connectivity within two sub-networks of the DMN: (i) an anterior sub-network, comprised of connections between all medial PFC regions in the DMN, and (ii) a posterior-to-anterior sub-network, comprised of connections between PCC regions and medial PFC regions. We compared the internal consistency reliability, test–retest reliability, and replicability of these sub-network averages to the full DMN average (Figure 2).

Internal consistency was adequate for connectivity within the anterior sub-network though lower for the posterior-to-anterior sub-network. Notably, although means and standard deviations for the sub-network averages replicated almost exactly across all samples, standard deviations were about double and internal consistency reliability was more variable across samples for the sub-network averages than the full network average (Figure 2). This was particularly notable for posterior-to-anterior connectivity, for which internal consistency reliability in Sample 3 was outside the 95% confidence interval of Sample 1, indicating a lack of replication. Test–retest reliability over eight weeks (available only in Sample 3) was moderate in the sub-network averages and comparable to the test–retest reliability of the full network average (Figure 2).

We also examined relationships with data quality (that is, number of censored time points due to spikes or motion). Although there was no relationship between data quality and the full DMN average (*r*=−0.09, *P*=0.10), poorer data quality (that is, more time points censored) was associated with lower sub-network averages (anterior sub-network *r*=−0.18, *P*=0.001; posterior-to-anterior sub-network *r*=−0.13, *P*<0.05).

### Normative data

Combining across all 322 healthy adult participants, average DMN connectivity in units of Fisher’s *Z* was 0.39 with a standard deviation of 0.07. Mean anterior sub-network connectivity was 0.63, with a standard deviation of 0.13. Mean posterior-to-anterior connectivity was 0.47, with a standard deviation of 0.14 (Table 3). We examined whether these averages differed by demographic features of age, gender and education (Supplementary Materials) and found a significant inverse relationship with age, driven by lower connectivity after age 50 years. Therefore, separate norms are provided in Table 3 for adults under (*n*=253) and over (*n*=69) age 50 years.

### Case example

A 39 year-old single male with major depressive disorder who presented for medication treatment as part of a randomized controlled trial^30^ provides an illustrative case example for how these norms may be applied. Average DMN connectivity was within normal limits (Pearson *r*=0.37, Fisher’s z=0.39; equal to the normative mean), however, while anterior sub-network connectivity was within normal limits (Pearson *r*=0.62, Fisher’s z=0.73; 0.7 SD above the normative mean), the posterior-to-anterior sub-network showed hypo-connectivity (Pearson *r*=0.20, Fisher’s z=0.20; 1.9 SD below the normative mean). Prior research suggests that a connectivity profile such as this is predictive of good antidepressant outcomes.^41, 42^ Indeed, this patient received Escitalopram 10 mg and was classified as a remitter after eight weeks of treatment, with 80% reduction in self-reported and 62% reduction in clinician-rated symptoms.

## Discussion

Our aim was to establish the proof of principle for a reliable summary metric that can quantify the intrinsic network function of an individual patient and allow it to be interpreted relative to healthy normative peers. Focusing here on the DMN, we found that an average of functional connectivity strength was more reliable than more complex topological properties, and satisfied our criteria for an ideal summary metric. This summary metric, in combination with normative data, provides a way to quantify and interpret intrinsic brain networks at the level of a single patient. This approach represents a significant step toward clinical translation of basic neuroscience insights.

While there is much to be gained by the application of topological properties to understanding brain networks, here our focus was on the most reliable and parsimonious way to quantify and interpret DMN functioning in order to facilitate clinical translation. Although the topological properties may be capturing meaningful moment-by-moment variation in DMN connectivity, perhaps associated with subtle alterations in mood or fatigue, our purpose here was to characterize more trait-like aspects of network function that may underlie risk for or recovery from clinical disorders.^24^ For this purpose a simple average appears optimal.

In general, we found that all three candidate metrics had good reproducibility of their means and standard deviations across three samples totaling 322 healthy adults (although reliability measures were not always consistent across samples for the topological properties). The normative mean of average DMN connectivity reported here also replicates the average DMN connectivity reported in a prior investigation.^43^ The adequate internal consistency and test–retest reliability of the average connectivity metric is consistent with the reliability of resting functional connectivity networks, including the DMN, found in other investigations.^40, 44^

In examining theoretically important sub-networks within the DMN, we found that greater regional specificity (and thus fewer connections included in the average) produced slightly poorer reliability, suggesting that taking into account all connections within a network may provide a more stable estimate of the network’s strength. Consistent with this notion, the test–retest reliability of the full DMN average was significantly greater than the test–retest reliability of connectivity between specific voxels.^40^ This does not mean that there is not meaningful information captured by greater regional or sub-network specificity. At the same time, consistent with prior investigations,^2, 22^ the DMN can be considered as a single coherent unit, and averaging across many connections within the network may provide a more stable and reliable measure of this unit’s function than any single connection.

We therefore present normative data for average connectivity in the DMN and its sub-networks. Although the sub-network averages demonstrated slightly poorer reliability and slightly greater relationships with fMRI data quality, we recognize they provide useful information for hypothesis generation (for example, suggesting that a certain aspect of the DMN is most responsible for overall hyper- or hypo-connectivity within the network, as in the case example presented here). Consistent with previous research on aging,^45^ we found that average connectivity dropped substantially after age 50 years, leading us to report normative data not only for the full sample, but also separately for adults older and younger than 50 years of age.

The norms presented here should be considered provisional, pending future investigations examining the impact of scanner model, scan acquisition parameters, data preprocessing algorithms, and demographic variables on average connectivity. Nevertheless they illustrate how a patient’s network function can be placed in context by expressing average network connectivity as a number of s.d. above or below the mean for the patient’s age group. This allows future research or clinical investigations to describe a patient or group of patients as within normal limits or abnormal, without needing to spend resources collecting data on a new (and likely small) sample of healthy comparison subjects.

As shown in the case example, a major benefit of a normed network summary metric is that it is easy to understand. By summarizing DMN connectivity into a single metric, it can be presented both as an effect size (Pearson *r*) and relative to a normative group (number of s.d. outside the normative mean). Furthermore, including both the overall DMN connectivity average and the sub-network averages allows for novel hypothesis generation of where an individual’s deficits may lie (as in the case example).

This approach highlights clear next steps for clinical translational research. Information about a patient’s pre-treatment network function can begin to inform patient care when the relationship between network function and treatment outcomes is also known. Thus, future research should identify what cut-point provides the best sensitivity and specificity to indicate the likelihood of response to a given treatment. This may include further exploration to identify the most reliable, valid, and clinically useful fMRI measures for both rest and task-evoked activity. Such research will need to explicitly consider cost-effectiveness and incremental validity of fMRI measures over other pieces of clinical data. Another crucial line of research will be to identify changes in network function resulting from a variety of treatment options.

Ultimately, when a clinician knows that a particular patient had substantially below average DMN connectivity (as in the case example), and that certain treatments increase DMN connectivity and/or can compensate for low DMN connectivity, then a targeted, individualized treatment strategy can emerge. Steps toward this type of individualized, normed connectivity-based approach to treatment are already underway for deep brain stimulation.^46^

### Limitations

The samples presented here are primarily Caucasian and entirely Australian, which potentially limits the applicability of the findings to other populations. There is no reason to suspect systematic differences in brain network function in different racial or ethnic groups, or based on country of origin. However, future work should include individuals from a wide range of backgrounds to confirm the applicability of the norms.

In all three samples presented here, the data were collected using the same model of scanner, the same acquisition parameters, and prepared with the same preprocessing algorithm. This uniformity is a strength in many respects. At the same time, an important line of future research will be to develop normative data for network function that takes variation in scanner model, acquisition parameters, and processing steps into consideration. Alternately, the development and widespread use of standardized acquisition and processing pipelines^47^ will greatly enhance the utility of normative data such as presented here.

The present data allow for an estimation of resting functional connectivity based on periods of rest within a series of tasks, as opposed to a stand-alone resting scan. These two procedures have been shown to yield remarkably similar results,^33^ and average connectivity values here were similar to those derived from stand-alone resting state scans.^43^ Furthermore, estimates of functional connectivity were remarkably consistent when computed within each task separately, as indicated by the internal consistency analysis. Regardless, future studies should compare our results to those using the more traditional stand-alone resting state scans.

### Conclusions and future directions

Overall, the present results suggest a roadmap for translating insights from basic neuroscience into clinical practice and highlight several concrete next steps. First, normative data for average connectivity should be expanded using larger, more generalizable samples (data collection for one such large sample of young adults is already ongoing^48^). This work should include an examination of scanner model, data acquisition parameters, and preprocessing algorithms, as discussed above. Second, the development of a reliable summary metric of network function may facilitate reverse translation from human research into animal models, allowing future research to probe the mechanisms underlying these complex systems. Third, our approach should be expanded to other clinically relevant brain networks, such as the salience, central executive, and reward networks.^2, 6^ Finally, studies should seek to relate normed metrics of network function to clinical variables, with a particular emphasis on profiles across multiple brain networks that map specifically to clinical profiles. Ultimately, to our knowledge, the present results represent a first step toward using intrinsic brain network analogously to normed neurocognitive and personality measures that are already in wide clinical use.

## Figures and Tables

**Table 1:** Description of participants in each sample

	*Sample 1*	*Sample 2*	*Sample 3*
Number of participants	126	129	67
Age (Mean (s.d.)), years	39.6 (12.8)	39.0 (13.1)	30.2 (12.6)
Gender (% female)	61.9%	62.0%	50.7%
Ethnicity (% Caucasian)	100%	100%	85.1%
Years of Education (Mean (s.d.))	14.6 (3.1)	14.4 (2.8)	14.8 (2.6)

**Table 2:** Comparison of three candidate metrics

	*Sample 1*	*Sample 2*	*Sample 3*
*Global clustering coefficient*			
Mean (SD)	0.82 (0.03)	0.83 (0.03)	0.82 (0.03)
Internal consistency: *α* [95% CI]	0.61 [0.47,0.71]	0.59 [0.44,0.71]	0.66 [0.45,0.79]
Test–retest: ICC [95% CI]	—	—	0.36 [0.12,0.59]

*Characteristic path length*			
Mean (SD)	1.40 (0.07)	1.41 (0.07)	1.38 (0.07)
Internal consistency: *α* [95% CI]	0.55 [0.41,0.68]	0.51 [0.31,0.65]	0.74^a^ [0.57,0.85]
Test–retest: ICC [95% CI]	—	—	0.45 [0.25,0.64]

*Average connectivity*			
Mean (SD)	0.39 (0.07)	0.40 (0.07)	0.39 (0.06)
Internal consistency: *α* [95% CI]	0.70 [0.55,0.79]	0.69 [0.55,0.82]	0.70 [0.53,0.83]
Test–retest: ICC [95% CI]	—	—	0.62 [0.46,0.75]

Abbreviations: CI, confidence interval; SD, standard deviation.

a Value outside 95% CI of Sample 1.

**Table 3:** Normative data for average functional connectivity

	*Mean*	*s.d.*	*25*th–*75*th *Percentile*
*Whole DMN*			
All Subjects (*N*=322)	0.39	0.07	0.34–0.43
Age≤50 (*N*=253)	0.40	0.07	0.36–0.45
Age>50 (*N*=69)	0.36	0.06	0.32–0.40

*Anterior sub-network*			
All Subjects (*N*=322)	0.63	0.13	0.54–0.71
Age≤50 (*N*=253)	0.64	0.13	0.56–0.72
Age>50 (*N*=69)	0.57	0.11	0.49–0.65

*Posterior-to-anterior sub-network*			
All Subjects (*N*=322)	0.47	0.14	0.36–0.57
Age≤50 (*N*=253)	0.48	0.14	0.39–0.58
Age>50 (*N*=69)	0.42	0.13	0.31–0.52

Abbreviation: DMN, default mode network.

## References

[bib1] Buckner RL, Andrews‐Hanna JR, Schacter DL. The brain's default network. Ann N Y Acad Sci 2008; 1124: 1–38. 18400922 10.1196/annals.1440.011

[bib2] Menon V. Large-scale brain networks and psychopathology: a unifying triple network model. Trends Cogn Sci 2011; 15: 483–506. 21908230 10.1016/j.tics.2011.08.003

[bib3] van den Heuvel MP, Hulshoff Pol HE. Exploring the brain network: A review on resting-state fMRI functional connectivity. Eur Neuropsychopharmacol 2010; 20: 519–534. 20471808 10.1016/j.euroneuro.2010.03.008

[bib4] Whitfield-Gabrieli S, Ford JM. Default Mode Network Activity and Connectivity in Psychopathology. Annu Rev Clin Psychol 2012; 8: 49–76. 22224834 10.1146/annurev-clinpsy-032511-143049

[bib5] Zhang D, Raichle ME. Disease and the brain's dark energy. Nature Reviews Neurology 2010; 6: 15–28. 20057496 10.1038/nrneurol.2009.198

[bib6] Williams LM. Precision psychiatry: a neural circuit taxonomy for depression and anxiety. Lancet Psychiatry 2016; 3: 472–480. 27150382 10.1016/S2215-0366(15)00579-9 PMC4922884

[bib7] Siegle GJ. Beyond depression commentary: wherefore art thou. Depression Clinic of Tomorrow? Clinical Psychology: Science and Practice 2011; 18: 305–310. 24634570 10.1111/j.1468-2850.2011.01261.x PMC3951918

[bib8] Dubois J, Adolphs R. Building a science of individual differences from fMRI. Trends Cogn Sci 2016; 20: 425–443. 27138646 10.1016/j.tics.2016.03.014 PMC4886721

[bib9] Ioannidis JP. Why most published research findings are false. PLoS Med 2005; 2: e124. 16060722 10.1371/journal.pmed.0020124 PMC1182327

[bib10] Huys QJ, Maia TV, Frank MJ. Computational psychiatry as a bridge from neuroscience to clinical applications. Nat Neurosci 2016; 19: 404–413. 26906507 10.1038/nn.4238 PMC5443409

[bib11] Daniels JK, McFarlane AC, Bluhm RL, Moores KA, Clark CR, Shaw ME et al. Switching between executive and default mode networks in posttraumatic stress disorder: Alterations in functional connectivity. J Psychiatry Neurosci 2010; 35: 258–267. 20569651 10.1503/jpn.090175 PMC2895156

[bib12] Garrity AG, Pearlson GD, McKiernan K, Lloyd D, Kiehl KA, Calhoun VD. Aberrant “default mode” functional connectivity in schizophrenia. Am J Psychiatry 2007; 164: 450–457. 17329470 10.1176/ajp.2007.164.3.450

[bib13] Stern ER, Fitzgerald KD, Welsh RC, Abelson JL, Taylor SF. Resting-state functional connectivity between fronto-parietal and default mode networks in obsessive-compulsive disorder. PLoS One 2012; 7: e36356. 22570705 10.1371/journal.pone.0036356 PMC3343054

[bib14] Calhoun V, Adali T, Pearlson G, Pekar J. A method for making group inferences from functional MRI data using independent component analysis. Hum Brain Mapp 2001; 14: 140–151. 11559959 10.1002/hbm.1048 PMC6871952

[bib15] van de Ven VG, Formisano E, Prvulovic D, Roeder CH, Linden DE. Functional connectivity as revealed by spatial independent component analysis of fMRI measurements during rest. Hum Brain Mapp 2004; 22: 165–178. 15195284 10.1002/hbm.20022 PMC6872001

[bib16] Zuo X-N, Kelly C, Adelstein JS, Klein DF, Castellanos FX, Milham MP. Reliable intrinsic connectivity networks: test–retest evaluation using ICA and dual regression approach. Neuroimage 2010; 49: 2163–2177. 19896537 10.1016/j.neuroimage.2009.10.080 PMC2877508

[bib17] Cole DM, Smith SM, Beckmann CF. Advances and pitfalls in the analysis and interpretation of resting-state FMRI data. Front Syst Neurosci 2010; 4: 8. 20407579 10.3389/fnsys.2010.00008 PMC2854531

[bib18] Fornito A, Zalesky A, Breakspear M. The connectomics of brain disorders. Nat Rev Neurosci 2015; 16: 159–172. 25697159 10.1038/nrn3901

[bib19] Rubinov M, Sporns O. Complex network measures of brain connectivity: Uses and interpretations. Neuroimage 2010; 52: 1059–1069. 19819337 10.1016/j.neuroimage.2009.10.003

[bib20] van den Heuvel MP, Sporns O. Network hubs in the human brain. Trends Cogn Sci 2013; 17: 683–696. 24231140 10.1016/j.tics.2013.09.012

[bib21] Gordon EM, Laumann TO, Adeyemo B, Huckins JF, Kelley WM, Petersen SE. Generation and evaluation of a cortical area parcellation from resting-state correlations. Cereb Cortex 2015; 26: 288–303. 10.1093/cercor/bhu239 PMC4677978 25316338

[bib22] Greicius MD, Krasnow B, Reiss AL, Menon V. Functional connectivity in the resting brain: A network analysis of the default mode hypothesis. Proc Natl Acad Sci USA 2003; 100: 253–258. 12506194 10.1073/pnas.0135058100 PMC140943

[bib23] Yeo BTT, Krienen FM, Sepulcre J, Sabuncu MR, Lashkari D, Hollinshead M et al. The organization of the human cerebral cortex estimated by intrinsic functional connectivity. J Neurophysiol 2011; 106: 1125–1165. 21653723 10.1152/jn.00338.2011 PMC3174820

[bib24] Fox MD, Greicius M. Clinical applications of resting state functional connectivity. Front Syst Neurosci 2010; 4: 19. 20592951 10.3389/fnsys.2010.00019 PMC2893721

[bib25] Broyd SJ, Demanuele C, Debener S, Helps SK, James CJ, Sonuga-Barke EJS. Default-mode brain dysfunction in mental disorders: A systematic review. Neurosci Biobehav Rev 2009; 33: 279–296. 18824195 10.1016/j.neubiorev.2008.09.002

[bib26] Rocca MA, Valsasina P, Absinta M, Riccitelli G, Rodegher ME, Misci P et al. Default-mode network dysfunction and cognitive impairment in progressive MS. Neurology 2010; 74: 1252–1259. 20404306 10.1212/WNL.0b013e3181d9ed91

[bib27] Henson RK. Understanding internal consistency reliability estimates: a conceptual primer on coefficient alpha. Measurem Eval Counsel Dev 2001; 34: 177.

[bib28] Shrout PE. Measurement reliability and agreement in psychiatry. Stat Methods Med Res 1998; 7: 301–317. 9803527 10.1177/096228029800700306

[bib29] Gatt JM, Korgaonkar MS, Schofield PR, Harris A, Clark CR, Oakley KL et al. The TWIN-E project in emotional wellbeing: Study protocol and preliminary heritability results across four MRI and DTI measures. Twin Research and Human Genetics 2012; 15: 419–441. 22856376 10.1017/thg.2012.12

[bib30] Williams LM, Rush AJ, Koslow SH, Wisniewski SR, Cooper NJ, Nemeroff CB et al. International Study to Predict Optimized Treatment for Depression (iSPOT-D), a randomized clinical trial: rationale and protocol. Trials 2011; 12: 1. 21208417 10.1186/1745-6215-12-4 PMC3036635

[bib31] Mulders PC, van Eijndhoven PF, Schene AH, Beckmann CF, Tendolkar I. Resting-state functional connectivity in major depressive disorder: a review. Neurosci Biobehav Rev 2015; 56: 330–344. 26234819 10.1016/j.neubiorev.2015.07.014

[bib32] Korgaonkar MS, Grieve SM, Etkin A, Koslow SH, Williams LM. Using standardized fMRI protocols to identify patterns of prefrontal circuit dysregulation that are common and specific to cognitive and emotional tasks in major depressive disorder: first wave results from the iSPOT-D study. Neuropsychopharmacology 2013; 38: 863–871. 23303059 10.1038/npp.2012.252 PMC3671994

[bib33] Korgaonkar MS, Ram K, Williams LM, Gatt JM, Grieve SM. Establishing the resting state default mode network derived from functional magnetic resonance imaging tasks as an endophenotype: A twins study. Hum Brain Mapp 2014; 35: 3893–3902. 24453120 10.1002/hbm.22446 PMC6869646

[bib34] Power JD, Mitra A, Laumann TO, Snyder AZ, Schlaggar BL, Petersen SE. Methods to detect, characterize, and remove motion artifact in resting state fMRI. Neuroimage 2014; 84: 320–341. 23994314 10.1016/j.neuroimage.2013.08.048 PMC3849338

[bib35] Power JD, Barnes KA, Snyder AZ, Schlaggar BL, Petersen SE. Spurious but systematic correlations in functional connectivity MRI networks arise from subject motion. Neuroimage 2012; 59: 2142–2154. 22019881 10.1016/j.neuroimage.2011.10.018 PMC3254728

[bib36] Andersson JL, Jenkinson M, Smith SNon-linear optimisationFMRIB technical report TR07JA1. University of Oxford FMRIB Centre: Oxford, UK, 2007.

[bib37] Yan C-G, Wang X-D, Zuo X-N, Zang Y-F. DPABI: data processing & analysis for (resting-state) brain imaging. Neuroinformatics 2016; 14: 339–351. 27075850 10.1007/s12021-016-9299-4

[bib38] Opsahl T. tnet: Software for analysis of weighted, two-mode, and longitudinal networks. R package 2007.

[bib39] Xia M, Wang J, He Y. BrainNet Viewer: a network visualization tool for human brain connectomics. PLoS One 2013; 8: e68910. 23861951 10.1371/journal.pone.0068910 PMC3701683

[bib40] Shehzad Z, Kelly AMC, Reiss PT, Gee DG, Gotimer K, Uddin LQ et al. The Resting Brain: Unconstrained yet Reliable. Cereb Cortex 2009; 19: 2209–2229. 19221144 10.1093/cercor/bhn256 PMC3896030

[bib41] Goldstein-Piekarski AN, Staveland B, Ball TM, Yesavage J, Korgaonkar MS, Williams LMIntrinsic functional connectivity predicts remission on antidepressants: a randomized-controlled trial to identify clinically applicable imaging biomarkers. Submitted Manuscript. 10.1038/s41398-018-0100-3 PMC5838245 29507282

[bib42] Dichter GS, Gibbs D, Smoski MJ. A systematic review of relations between resting-state functional-MRI and treatment response in major depressive disorder. J Affective Dis 2015; 172: 8–17. 10.1016/j.jad.2014.09.028 PMC4375066 25451389

[bib43] Whitfield-Gabrieli S, Thermenos HW, Milanovic S, Tsuang MT, Faraone SV, McCarley RW et al. Hyperactivity and hyperconnectivity of the default network in schizophrenia and in first-degree relatives of persons with schizophrenia. Proc Natl Acad Sci USA 2009; 106: 1279–1284. 19164577 10.1073/pnas.0809141106 PMC2633557

[bib44] Damoiseaux JS, Rombouts SARB, Barkhof F, Scheltens P, Stam CJ, Smith SM et al. Consistent resting-state networks across healthy subjects. Proc Natl Acad Sci USA 2006; 103: 13848–13853. 16945915 10.1073/pnas.0601417103 PMC1564249

[bib45] Sambataro F, Murty VP, Callicott JH, Tan H-Y, Das S, Weinberger DR et al. Age-related alterations in default mode network: Impact on working memory performance. Neurobiol Aging 2010; 31: 839–852. 18674847 10.1016/j.neurobiolaging.2008.05.022 PMC2842461

[bib46] Widge AS, Ellard KK, Paulk AC, Basu I, Yousefi A, Zorowitz S et al. Treating refractory mental illness with closed-loop brain stimulation: Progress towards a patient-specific transdiagnostic approach. Exp Neurol 2017; 287: 461–472. 27485972 10.1016/j.expneurol.2016.07.021

[bib47] Gorgolewski KJ, Alfaro-Almagro F, Auer T, Bellec P, Capota M, Chakravarty MM et al. BIDS Apps: improving ease of use, accessibility and reproducibility of neuroimaging data analysis methods. PLoS Comput Biol 2017; 13: e1005209. 28278228 10.1371/journal.pcbi.1005209 PMC5363996

[bib48] Van Essen DC, Smith SM, Barch DM, Behrens TE, Yacoub E, Ugurbil K et al. The WU-Minn Human Connectome Project: an overview. Neuroimage 2013; 80: 62–79. 23684880 10.1016/j.neuroimage.2013.05.041 PMC3724347

