# Supplementary material for: Quantifying person-level brain network functioning to facilitate clinical translation
Source: Transl Psychiatry. 2017 Oct 17;7(10):e1248–. doi: 10.1038/tp.2017.204 (PMC5682602; doi:10.1038/tp.2017.204)
Supplement: Supplementary Information [file tp2017204x1.docx]

**Supplemental Materials**

In generating the topological properties (global clustering coefficient and characteristic path length) we used a cut-off of 70% density, as this was the lowest density resulting in 100% of participants in Sample 1 with fully connected graphs. We also constructed graphs using 75% and 80% density to explore whether this parameter substantially alters the results (Supplemental Table 1).

We also explored relationships between average DMN connectivity and demographic variables (age, gender, and years of education). Race and ethnicity was not examined due to very low numbers of non-White participants (3% of participants). There is no theoretical reason to believe there will be differences in connectivity across racial groups; future research should confirm consistency of the normative data across these populations.

There was a significant inverse correlation between average connectivity and age for the full DMN average (r = -.26, p < .001), anterior sub-network average (r = -.20, p < .001), and posterior-to-anterior sub-network average (r = -.15, p = .006). This is consistent with prior research showing reduced functional connectivity with increasing age (*1*). Visual inspection of the data revealed a drop in connectivity particularly after 50 years of age (Supplemental Figure 1). There were no differences between men and women on average connectivity (all ps > .25). There were also no relationships between average connectivity and years of education (all ps > .07).

*Supplemental Table 1. Comparison of density thresholds*

|  | 70% density | 75% density | 80% density |
| --- | --- | --- | --- |
| Global Clustering Coefficient |  |  |  |
| Mean (SD) | 0.82 (0.03) | 0.85 (0.03) | 0.87 (0.02) |
| Internal Consistency: α [95% CI] | 0.61 [.52, .68] | 0.61 [.53, .68] | 0.61 [.52, .69] |
| Test-Retest: ICC [95% CI] | 0.36 [.12, .59] | 0.38 [.13, .59] | 0.36 [.13, .55] |
| Characteristic Path Length |  |  |  |
| Mean (SD) | 1.40 (0.07) | 1.34 (0.07) | 1.28 (0.07) |
| Internal Consistency: α [95% CI] | 0.58 [.48, .66] | 0.59 [.49, .66] | 0.60 [.50, .67] |
| Test-Retest: ICC [95% CI] | 0.45 [.25, .64] | 0.46 [.24, .64] | 0.46 [.23, .65] |

Note. Combining across all samples, except test-retest (only available in Sample 3). SD = standard deviation; CI = confidence interval; ICC = intra-class correlation coefficient.

| Supplemental Figure 1 |
| --- |
| 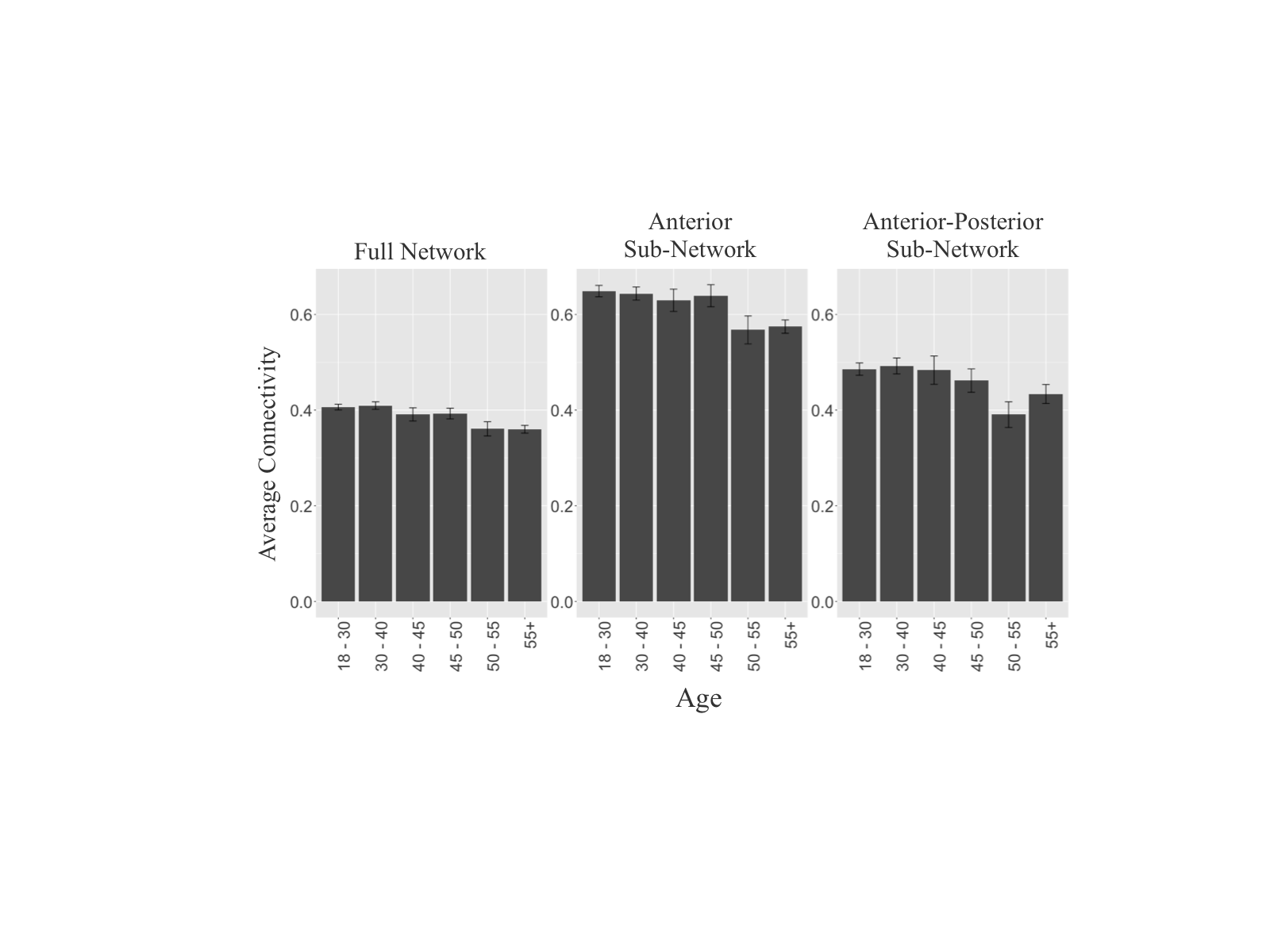 |
|  |

**References**

1. F. Sambataro, V. P. Murty, J. H. Callicott, H.-Y. Tan, S. Das, D. R. Weinberger, V. S. Mattay, Age-related alterations in default mode network: Impact on working memory performance. *Neurobiol. Aging* **31**, 839-852 (2010).
